# Image-guided biopsy of breast lesions—when to use what biopsy technique

**DOI:** 10.1186/s13244-025-02084-5

**Published:** 2025-09-25

**Authors:** Wendelien B. G. Sanderink, Julia Camps-Herrero, Alexandra Athanasiou, Henrique L. Couto, Kirti Mehta, Popat Palak Bhavesh Thakkar, Pooja Jagmohan, Sara E. Vázquez-Manjarrez, Seigo Nakamura, Jelle Wesseling, Ritse M. Mann

**Affiliations:** 1 Department of Medical Imaging, Radboud University Medical Center, Nijmegen, The Netherlands; 2 Breast Health, Ribera Salud Hospitals, Valencia, Spain; 3 Breast Imaging Department, MITERA Hospital, Athens, Greece; 4 Redimama-Redimasto Breast Unit, Belo Horizonte, Brazil; 5 Department of Radiology, Imaging Associates/Eastern Health, Melbourne, Victoria Australia; 6 Department of Radiology, Tata Memorial Hospital and Homi Bhabha National Institute, Mumbai, India; 7 Department of Diagnostic Imaging, National University Hospital, Singapore, Singapore; 8 Department of Diagnostic Radiology, Yong Loo Lin School of Medicine, National University of Singapore, Singapore, Singapore; 9 Department of Breast Imaging and Interventional Radiology, National Institute of Health Sciences and Nutrition Salvador Zubirán, Mexico City, Mexico; 10 Division of Breast Surgical Oncology, Department of Surgery, Showa University School of Medicine, Tokyo, Japan; 11 Institute for Clinical Genetics and Genomics, Showa University, Tokyo, Japan; 12 Division of Molecular Pathology, Netherlands Cancer Institute, Amsterdam, The Netherlands; 13 Department of Pathology, The Netherlands Cancer Institute – Antoni van Leeuwenhoek, Amsterdam, The Netherlands; 14 Department of Pathology, University Medical Centre, Leiden, The Netherlands; 15 Early Detection Center, Netherlands Cancer Institute, Amsterdam, The Netherlands; 16 Department of Radiology, The Netherlands Cancer Institute, Amsterdam, The Netherlands

**Keywords:** Breast cancer, Needle biopsy, Consensus

## Abstract

**Abstract:**

In recent years, minimally invasive diagnostic options for breast lesions have expanded, but consensus on optimal biopsy techniques and imaging combinations remains lacking. This study, driven by an adapted RAND-UCLA Appropriateness Method and insights from eight experts in breast biopsy from across the world, aims to create consensus for selecting biopsy techniques. Highlighted findings suggest Vacuum-Assisted Biopsy (VAB) for lesions visible exclusively at mammography/tomosynthesis (with or without contrast enhancement) or MRI. Core-needle biopsy (CNB) takes precedence for masses over 5 mm visible under US. The selection of other biopsy techniques during US-guided procedures depends on lesion type, size, and sampling indication. VAB is preferred for smaller masses (< 5 mm), complex cystic and solid lesions with small solid parts, small intraductal masses, architectural distortions, and calcifications visible on US. In re-biopsy scenarios for inconclusive findings or high-risk lesions, the panel suggests two VAB extensions: Extended Vacuum-Assisted Biopsy (EVAB) for unambiguous lesion classification and Vacuum-Assisted Excision (VAE) for complete lesion removal. Furthermore, the panel provides detailed input on how to handle specific cases, such as re-biopsy for lobular neoplasia, flat epithelial atypia and atypical ductal hyperplasia. Surgical excision is advised for DCIS and benign or borderline phyllodes tumors found through initial CNB or VAB. In conclusion, an international expert group formulated recommendations on diagnostic breast biopsies under image guidance, aiming to ensure accurate diagnosis worldwide by providing practical advice on needle selection and biopsy approach.

**Key Points:**

- Evidence-based literature on the preferred biopsy technique and imaging combination for the diagnosis of breast lesions is sparse, and a general consensus is not available.
- The selection of biopsy technique for different image-guided procedures depends on lesion type, size, and sampling indication.
- This international expert panel consensus statement addresses standard approaches for varying biopsy indications.

**Graphical Abstract:**

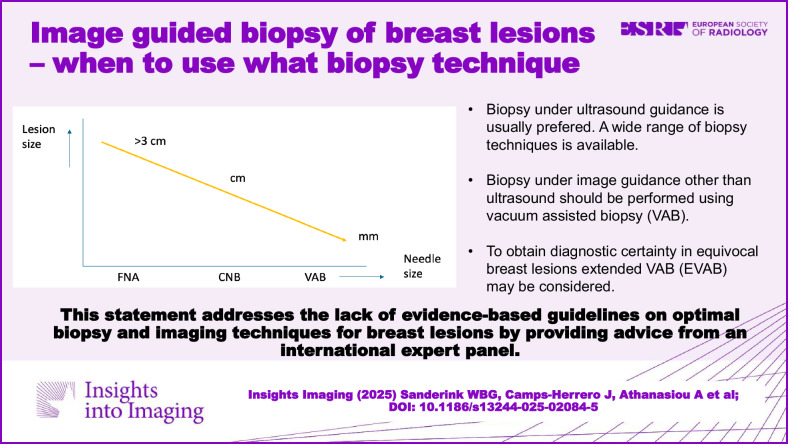

## Introduction

Percutaneous image-guided needle biopsy of the breast has become the standard of care for the diagnosis of suspicious breast lesions. It is an accurate diagnostic procedure with fewer complications, lower cost and better cosmetic outcomes compared to diagnostic surgical biopsy. Current methods for diagnosis include fine-needle aspiration (FNA), core-needle biopsy (CNB) and vacuum-assisted biopsy (VAB), each with specific advantages and limitations [1, 2]. These techniques can be performed under the guidance of different imaging techniques. The choice for mammography/tomosynthesis, with or without contrast enhancement (e.g., contrast-enhanced mammography (CEM)), ultrasound (US), or magnetic resonance imaging (MRI) guidance is based upon lesion visibility, location and expected difficulty and burden of the procedure [1]. Although every practitioner of breast biopsies should be aware of the advantages and limitations of biopsy and imaging techniques, evidence-based literature discussing which biopsy technique and imaging combination is to be preferred for the diagnosis of breast lesions is sparse, and a general consensus is not available. This article provides an expert consensus for biopsy of breast lesions and re-biopsy in case of radiological-pathological discordance or findings of uncertain malignant potential.

## Materials and methods

The RAND/UCLA Appropriateness Method (RAM) was used to obtain consensus in an expert panel on the use of biopsy techniques in combination with imaging techniques for diagnosis and re-biopsy of breast lesions. RAM is a modified Delphi method that, unlike the original Delphi, allows panelists to discuss their judgments in person. Panelists rate each question twice, in a two-round ‘modified Delphi’ process [3].

Email invitations were sent to persons identified as regional/global key opinion leaders in breast biopsy based upon scientific output, presence at loco-regional and international conferences, participation and membership in scientific and educational breast imaging bodies and discussion with local breast imaging specialists. The final selection of participants was made following discussions between the study leads and the sponsor. Medical professions differed due to varying customs across the world. All approached persons possess extensive experience in delivering lectures and workshops on breast biopsies at both national and international conferences. Eight were willing to participate: 6 radiologists, 1 surgeon, 1 mastologist (a specialized physician focusing on the study, diagnosis, and treatment of breast diseases). All the panel members also had extensive clinical experience in various breast biopsy techniques. Particular emphasis was placed on their engagement in local policy decision-making processes, specifically in the context of selecting biopsy methods. Their direct involvement in determining the most appropriate biopsy techniques at the regional level ensured that the panel provided a well-informed and practice-oriented perspective. Characteristics of the voting panel members are summarized in Table 1. A summary of the different imaging modalities used for screening, diagnosis and staging by the panel members can be found in Appendix 1 (Supplementary Table 1, page 2).

**Table 1:** Characteristics of the voting panel members

Characteristics	*N*
Discipline	
Radiologist	6
Surgeon	1
Mastologist	1
Region	
Europe	2
Latin America	2
Asia-Pacific	4
Median (range) years of experience with breast biopsies	18 (10–25)
Median (range) number of breast biopsies per year	225 (150–831)

The panel included two non-voting observers who presented the data, controlled the discussion and were in charge of drafting the manuscript. One participant chaired the meeting. An independent breast pathologist critically reviewed and revised the manuscript. Voting participants presented a reference deck with local and regional breast cancer statistics from their hospital and region. All panel members were asked to act as a regional representative including the viewpoints of their local peers. A 2-day face-to-face meeting was held in December 2022 in Aalst, Belgium. BD (Becton, Dickinson and Company) facilitated the meeting, but did not participate in the actual discussion, nor did they have any influence on the results. To inform the panel, a non-systematic literature review was presented by a non-voting participant, summarizing key available scientific evidence regarding biopsy and imaging techniques for breast lesion diagnosis and re-biopsy, see Appendix 2 (Supplementary Material, page 3) for a summary.

To understand the level of variability in the choices for a specific combination of a biopsy method and an imaging method, the first day involved brainstorming exercises. Participants used post-its on a table displaying biopsy methods (*y*-axis) and imaging techniques (*x*-axis), indicating their preferred combinations for various situations. This yielded baseline data on items for which consensus was already present and items that required further discussion. All divergent suggestions were discussed within the panel.

Based on the discussed items a questionnaire was constructed at the end of day 1 by the non-voting members in cooperation with the meeting chair. This questionnaire included the choice of biopsy technique by imaging modality, and the choice for different types of lesions under ultrasound guidance in both the primary diagnostic setting and after initial biopsy-based diagnosis of a lesion of uncertain malignant potential. Each panelist was also asked to rate different facilitators and barriers on a 5-point scale (1: not important, 5: very important).

The developed questionnaire used for the consensus procedure is provided in Appendix 3 (Supplementary Material, page 4). Two voting rounds occurred on day 2. After the first round, votes were summarized, and the panel discussion focused on items for which no consensus had been achieved. During the discussion, panelists found some items to be inadequately phrased, in which case the items were rephrased for the second voting round or discarded. Following the discussion of all questions, the panelists were asked to vote again.

Consensus was achieved when at least 75% of participants agreed after the second voting round on the combination of biopsy technique and image guidance in a specific situation. Majority agreement was achieved when 50–75% agreed.

Final statements were collected following the online written documentation of the consensus meeting. Statements were sent to all participants, and further adaptations could be proposed (based on written or email correspondence). Following the meeting, regular email correspondence was maintained with all participants to ensure the validity and relevance of the statements. In these exchanges, all members confirmed their continued agreement with the consensus. A final draft of the paper was prepared after an online panel discussion. Written agreement was obtained from all participants.

## Results

### Used definitions

Following initial discussion, biopsy methods were defined as follows:

### Fine-needle aspiration

FNA is a technique in which a thin needle is inserted with periodical inward-outward movements in a lesion to aspirate cells or fluid. FNA does not yield sufficient tissue to obtain a histological diagnosis. FNA can be performed under local anesthesia, but this is often not necessary. For FNA the panel recommends the use of a needle size up to approximately 20 gauge (except when aspirating abscesses).

### Core-needle biopsy

CNB obtains a small sample (core) of tissue from the biopsied region, which can be used for histological classification and molecular subtypes of lesions. CNB needles, varying between 18 and 12 gauge in size, can be used for diagnostic breast biopsies. Sample length ranges between 1 and 2.5 cm. The panel recommends the use of a 14-gauge needle in most situations. Although a minimum of three cores is commonly advised for a reliable breast lesion diagnosis, the panel states that the number of samples needs to be based on the quality instead of the quantity of the samples.

### Vacuum-assisted biopsy

VAB is a CNB technique using vacuum suction to obtain more tissue than regular CNB. VAB is performed with larger needles that range in size from 12 to 7 gauge. The minimal number of samples that should be obtained with VAB depends on the needle size [4]. The panel recommends that when using a 9-gauge needle, at least six samples are obtained. When needles of other sizes are used, an equivalent minimum of tissue should be removed [4].

### Extended vacuum-assisted biopsy

The panel recommends using the term Extended Vacuum-Assisted Biopsy (EVAB) for a VAB procedure with an increased number of tissue samples for diagnosis. The purpose is to obtain a reliable histological diagnosis, not to remove an entire lesion. The number of tissue samples should correspond to the removal of approximately 4 g of tissue [5–7]. This equals, on average, 18 samples with a 10-gauge needle or 12 samples with a 7-gauge needle. However, the procedure can be stopped earlier if the lesion is visually completely removed. Although this procedure does not aim to remove lesions entirely, complete excision might occur for small lesions (5–10 mm).

### Vacuum-assisted excision

The aim of vacuum-assisted excision (VAE) is to remove the lesion entirely. It can be used as a therapeutic method for benign lesions and lesions of uncertain malignant potential, in line with the British National Institute for Health and Care Excellence (NICE) recommendation [8]. Samples should be obtained until the lesion is visually removed. The number of samples is limited only by the location of the lesion and the size of the breast. In general, the larger the needle, the easier complete lesion removal is achieved.

### General suggested standard approaches

In principle, tissue sampling under US guidance is always the first choice for breast lesions.

In US-visible masses, CNB is usually the first choice (consensus: 75% of panel). Although FNA is easy and cheap, it has lower sensitivity and specificity and is associated with a high underestimation rate and false positive findings. Moreover, in malignant lesions, additional biopsy techniques are necessary to obtain a clear diagnosis, as FNA does not differentiate in situ from invasive tumors, nor provides receptor status. Accordingly, the use of FNA for the diagnosis of US-visible lesions is generally discouraged. However, in centers with limited resources, FNA may be a residual option to reduce costs and expedite pathological reporting, particularly when performed in close collaboration with a dedicated pathologist to improve accuracy. VAB is a valid alternative to CNB in US-guided biopsies, but is more expensive and associated with more complications. Consequently, VAB should only be considered in specific situations as a first-line diagnostic biopsy method (see section below).

In lesions only visible on mammography, DBT or CEM, VAB is always the first choice (consensus: 100% of panel). It provides more tissue, resulting in a higher sensitivity and specificity and lower underestimation rate in comparison with FNA and CNB [9]. CNB is only an alternative in case VAB is not available or possible. In case of a large area with calcifications, it is recommended to perform two VAB biopsies at different places instead of one large biopsy to document the extent and reduce the risk of underestimation of an invasive carcinoma [10].

For lesions only visible on MRI, VAB is likewise recommended (consensus: 100% of panel). If VAB is not available, opt for an MRI-guided localization and surgical excision. In certain cases and depending on the level of suspicion and clinical context, a short-interval follow-up may be an acceptable alternative when neither CEM- nor MRI-guided biopsy is available [11].

### Suggested approaches in US-visible lesions

Although CNB is the first choice in US-visible masses, incidentally, other biopsy techniques need to be considered. This is dependent on lesion type, size and indication of tissue sampling. Specific lesion categories and indications for which recommendations might deviate from the general CNB recommendation when biopsy is performed under ultrasound are discussed below.

Table 2 provides an overview of approaches for specific US-visible lesions.

**Table 2:** Summary of statements and results of panel voting for US-visible lesions

Lesion type	Consensus	Results of panel voting	Comments
Masses < 5 mm	Yes	VAB: 100%	VAB is recommended due to the relatively high likelihood of sampling errors with CNB in these lesions. As it is likely that the lesion will be completely removed with VAB, placement of a marker at the biopsy cavity is essential.
Masses > 5 mm	Yes	CNB: 87.5% VAB: 12.5%	CNB is recommended for larger masses (> 5 mm), incidentally VAB may be considered.
Simple and complicated cyst	Yes	FNA: 100%	In symptomatic patients, the use of FNA is recommended to relieve the symptoms. In asymptomatic patients sampling is not necessary.
Complex cystic and solid lesion with small (< 5 mm) solid part(s)	Yes	VAB: 87.5% EVAB: 12.5%	VAB is recommended, due to the higher chance of underestimation and the risk of non-visualization of the solid part after rupture of the cyst. Just as with small masses, placement of a marker at the biopsy cavity is essential.
Complex cystic and solid lesion with larger (> 5 mm) solid part(s)	No	CNB: 50% VAB: 25% EVAB: 12.5% Surgical excision: 12.5%	Possibility to do CNB, but only if it is safe and the solid component is big. Regardless of the approach, if the lesion collapses during biopsy, a post-biopsy marker should be placed.
Small intraductal mass (< 5 mm)	Yes	VAB: 100%	VAB is recommended, due to the fact that it is a small mass it is likely to be removed completely with this procedure, with an excision as consequence. Alternatively, when opting for a CNB in intraductal lesions, be aware of the likelihood of underestimation. It is important that a sufficient number of samples are obtained.
Architectural distortion	Yes	VAB: 87.5% Surgical excision: 12.5%	For suspicious architectural distortion, typically presenting as a non-mass lesion, as will be defined in the upcoming BI-RADS lexicon, VAB is recommended, independent of the size of the architectural distortion. Due to the many benign and malignant entities that can cause architectural distortion, it is important that a sufficient amount of tissue is obtained to reduce the likelihood of underestimation and misdiagnosis. It can be considered to perform an EVAB directly.
Calcifications	Yes	VAB: 87.5% VAE: 12.5%	The panel highly recommends specimen radiography to confirm that calcifications are present in the tissue sample. The placement of a post-biopsy marker is mandatory in these cases.
Lymph nodes	No	FNA: 50% CNB: 50%	Use FNA only for confirmation of malignancy. If the pathological results from the biopsy have influence on the choice of treatment it is better to choose CNB.
Abscess	Yes	FNA: 100%	It is advised to use a relatively large needle, as abscess contents can be very viscous and are not easily removed through a thin needle. Abscess drainage should be combined with antibiotic treatment. Repeated aspiration may be required before complete resolution is achieved. With ultrasound, the progress of the abscess and the response to therapy can be monitored. In case the abscess recurs multiple times, a CNB might be considered to rule out a non-responding mastitis (e.g., granulomatous mastitis or inflammatory breast cancer).
Mastitis (not responding to antibiotics treatment)	Yes	CNB: 100%	Cores should be obtained from the inflamed area, or in case of inflammation of the entire breast, from multiple areas, to exclude inflammatory breast cancer.

*CNB* core-needle biopsy, *EVAB* extended vacuum-assisted biopsy, *FNA* fine-needle aspiration, *VAB* vacuum-assisted biopsy

### Small masses (< 5 mm)

For diagnostic biopsy of small masses (< 5 mm), VAB is recommended by the panel (consensus: 100% of panel), due to the relatively high likelihood of sampling errors with CNB in these lesions. As it is likely that the lesion will be completely removed with VAB, placement of a marker at the biopsy cavity is essential.

### Larger masses (> 5 mm)

The panel recommends CNB for larger masses (> 5 mm) visible under ultrasound (consensus: 87.5% of panel). Incidentally, VAB may be considered (recommended by one panelist, 12.5%).

### Simple and complicated cyst

The panel recommends FNA for simple and complicated cysts in symptomatic patients to relieve the symptoms (consensus: 100% of panel). In asymptomatic patients, sampling is not necessary.

### Complex cystic and solid lesions with small (< 5 mm) solid part(s)

The panel recommends VAB (consensus: 87.5% of panel) for complex cystic and solid breast lesions with a limited solid component, due to the higher chance of underestimation and the risk of non-visualization of the solid part after rupture of the cyst. Just as with small masses, placement of a marker at the biopsy cavity is essential.

### Complex cystic and solid lesions with larger (> 5 mm) solid part(s)

For biopsy of complex cystic and solid lesions with large solid parts, no consensus was reached. Half of the panelists (50%) recommended CNB for such complex cystic and solid lesions. Alternatively, VAB, EVAB or excision is proposed. Regardless of the approach, if the lesion collapses during biopsy, a post-biopsy marker should be placed.

### Small intraductal mass (< 5 mm)

The panel recommends VAB for biopsy of small intraductal masses (consensus: 100% of panel). Due to the fact that it is a small mass, it is likely to be removed completely with this procedure, with an excision as consequence. Alternatively, when opting for a CNB in intraductal lesions, be aware of the likelihood of underestimation. It is important that a sufficient number of samples are obtained.

### Architectural distortion

For suspicious architectural distortion that is visible under ultrasound, typically presenting as a non-mass lesion, as will be defined in the upcoming BI-RADS lexicon, VAB is recommended (consensus: 87.5% of panel), independent of the size of the architectural distortion. Due to the many benign and malignant entities that can cause architectural distortion, it is important that a sufficient amount of tissue is obtained to reduce the likelihood of underestimation and misdiagnosis. It can be considered to perform an EVAB directly.

### Calcifications

For biopsy of ultrasound visible calcifications, VAB is recommended (consensus: 87.5% of panel). The panel highly recommends specimen radiography to confirm that calcifications are present in the tissue sample. The placement of a post-biopsy marker is mandatory in these cases.

### Lymph nodes

For tissue sampling of lymph nodes consensus was not reached. 50% of the panel preferred FNA, and 50% preferred CNB. In general, when it is only important to prove that a lymph node is metastatic involved by the breast cancer, FNA is useful. When the pathological results from biopsy have an influence on the diagnosis and treatment, it is better to choose CNB, as sensitivity is higher and permits better characterization of the molecular profile. In view of recently published and ongoing trials on omitting axillary surgery (SOUND [12], BOOG 2013-08 [13], NEONOD 2 [14]) in clinically node-negative patients, it is better to opt for CNB due to the lower likelihood of underestimation.

### Abscess

The panel recommends FNA to drain abscesses (consensus: 100% of panel). It is advised to use a relatively large needle, as abscess contents can be very viscous and are not easily removed through a thin needle. Abscess drainage should be combined with antibiotic treatment. Repeated aspiration may be required before complete resolution is achieved. With ultrasound, the progress of the abscess and the response to therapy can be monitored. In case the abscess recurs multiple times, a CNB might be considered to rule out a non-responding mastitis (e.g., granulomatous mastitis or inflammatory breast cancer).

### Mastitis not responding to antibiotic treatment

CNB is recommended in patients with mastitis that does not respond to antibiotic therapy (consensus: 100% of panel). Cores should be obtained from the inflamed area, or in case of inflammation of the entire breast from multiple areas, to exclude inflammatory breast cancer.

### Re-biopsy and minimally invasive intervention of US-visible lesions

During the initial brainstorm and subsequent discussion, it was acknowledged that in a number of situations, a second intervention after initial biopsy is required, either because the lesion is insufficiently sampled to come to a final diagnosis or because the lesion should be removed.

The specific situations and corresponding panel suggestions are listed below and are summarized in Table 3.

**Table 3:** Summary of statements and results of panel voting for re-biopsy of US-visible lesions

Reason for re-biopsy	Consensus	Results of panel voting	Comments
Proven benign lesion that the patient wants removed (< 3 cm)	Yes	VAE: 100%	VAE is recommended, with surgical excision as a backup procedure. Discuss the advantages and disadvantages with the patient to receive a well-informed consent.
Proven benign lesion that the patient wants removed (> 3 cm)	No	Surgical excision: 50% VAE: 50%	Surgical excision is recommended, with VAE as a backup procedure. Discuss the advantages and disadvantages with the patient to receive a well-informed consent.
Radiology-Pathology discordance on CNB	No	VAB: 62.5% EVAB: 37.4%	Repeat CNB should only be considered in the case of a fibroadenoma.
Radiology-Pathology discordance on VAB	No	EVAB: 25% VAE: 25% Surgical excision: 50%	VAE should only be considered for women at average risk.
High-risk (B3) lesion on CNB	No	VAB: 25% EVAB: 50% VAE: 25%	
High-risk (B3) lesion on VAB			
Lobular neoplasia	Yes	EVAB: 75% VAE: 25%	Follow-up is recommended if it is an incidental finding
Flat epithelial atypia	Yes	EVAB: 75% VAE: 25%	Follow-up is recommended if it is an incidental finding
ADH	No	EVAB: 62.5% Surgical excision: 37.5%	
Complex sclerosing lesion	No	Follow-up: 12.5% EVAB: 62.5% VAE: 25%	
Papillary lesion	No	EVAB: 62.5% VAE: 25% Surgical excision: 12.5%	Follow-up is recommended if the lesion is small and without any atypia.
Benign phyllodes tumor	No	EVAB: 25% VAE: 12.5% Surgical excision: 62.5%	For small (< 2 cm) benign phyllodes tumors, VAE can be considered. For larger (> 2 cm) benign phyllodes tumors, surgical excision is recommended.
Borderline phyllodes tumor	Yes	Surgical excision: 100%	
DCIS on CNB	Yes	VAB: 12.5% Surgical excision: 87.5%	EVAB can be considered for staging.
DCIS on VAB	Yes	Surgical excision: 100%	

*ADH* atypical ductal hyperplasia, *CNB* core-needle biopsy, *DCIS* ductal carcinoma in situ, *EVAB* extended vacuum-assisted biopsy, *VAB* vacuum-assisted biopsy, *VAE* vacuum-assisted excision

### A proven benign lesion that a patient wants to have removed

The most important step is to discuss the advantages and disadvantages of any procedure with the patient when she requests the removal of a proven benign lesion. The length of the procedure, the potential complications and the cosmetic results should be discussed. It is essential to receive a well-informed consent in this setting.

For benign lesions smaller than 3 cm, VAE is recommended by the panel (consensus: 100% of panel), with surgical excision as a backup procedure. For lesions bigger than 3 cm, both surgical excision and VAE can be considered, also depending on lesion location and size of the breast.

### Radiological-pathological discordance on initial biopsy

For radiological-pathological discordance after CNB, consensus was not reached. Most members of the panel advised the use of VAB (majority agreement: 62.5% of panel), whereas EVAB is regarded as an alternative. Repeat CNB is discouraged, unless the lesion is likely a fibroadenoma, in which case the lesion might just have been missed, and a diagnosis may be achieved by more successful targeting. When radiological-pathological discordance is present after VAB, EVAB, VAE or surgical excision were regarded as valid options, but consensus was not reached.

### Breast lesions of uncertain malignant potential

Lesions of uncertain malignant potential, also known as high-risk lesions or B3 lesions, represent a heterogeneous group of abnormalities with a variable but low risk of associated malignancy [15, 16]. Statements below are applicable only for women at average risk. For high-risk women surgical excision of high-risk lesions is generally advised.

When lobular neoplasia (atypical lobular hyperplasia (ALH) and classic lobular carcinoma in situ (LCIS)), or flat epithelial atypia (FEA) is an incidental finding on CNB, a re-biopsy using EVAB was the most common suggestion (majority agreement: 50% of panel), regular VAB (25%) and VAE (25%) are regarded as alternatives also depending on the underlying histology. Consensus was not reached. When the diagnosis follows upon biopsy of a lesion visible on US or when atypical ductal hyperplasia (ADH) is detected in the biopsy specimen, a re-biopsy with EVAB is always recommended.

For incidental findings of lobular neoplasia and flat epithelial atypia on VAB follow-up might be sufficient, alternatively, EVAB can be considered (consensus: 75% of panel).

For complex sclerosing lesions, including radial scars, with or without ductal atypia, seen on US, a re-biopsy with EVAB is recommended after an initial CNB or VAB (majority agreement: 62.5% of panel). If this EVAB is again negative for malignancy, follow-up is sufficient.

For large papillary lesions (> 1.5 cm), with or without ductal atypia, and for small papillary lesions (< 1.5 cm) with ductal atypia, the panel recommends a re-biopsy with EVAB to ensure complete evaluation of the lesion [17–19]. In small lesions (< 1.5 cm) without any ductal atypia on CNB or VAB, follow-up can also be considered (majority agreement: 62.5% of panel) [18, 19].

For phyllodes tumors, benign or borderline, found with initial CNB or VAB, surgical excision is advised (majority agreement: 62.5% of panel). Only for small (< 2 cm) benign phyllodes tumors, a VAE can be considered [20].

### DCIS

For ductal carcinoma in situ (DCIS) found with initial CNB or VAB, surgical excision is recommended by 87.5% of the panel for CNB and 100% for VAB findings, respectively. Additional imaging, with MRI or CEM, or additional sampling with VAB, is only advised if it affects treatment decisions. Especially when DCIS is initially found with CNB, there is a higher chance of underestimation, and additional imaging or sampling should be considered. If the lesion is visible as a mass or multiple masses, there is a higher chance of underestimation, and the treatment is more often influenced by additional sampling. This is accordingly advised.

## Facilitators and barriers

Defined facilitators and barriers are listed in Table 4. The table includes the mean score and range of responses gathered from the voting rounds, along with comments derived from the discussion.

**Table 4:** Results of panel voting for facilitators and barriers for the implementation of a biopsy technique

Facilitators and barriers	Mean score [range]^†^	Comments
Sensitivity of the biopsy technique	5.0 [5]	
Minor complications (no subsequent treatment needed)	2.3 [1–4]	Only important to take into account for specific patients, such as adolescent, pregnant or lactating women.
Major complications (require intervention)	4.1 [3–5]	
Cosmetics	3.5 [2–5]	A good diagnosis is always more important.
Patient comfort	3.8 [3–5]	
Procedure time	3.6 [3, 4]	
Costs	4.3 [3–5]	
Possibility to remove the entire lesion	4.1 [4, 5]	
Experience of the radiologist	4.8 [4, 5]	
Patient factors (e.g., anticoagulation)	3.9 [3–5]	

^†^ Averaged across all eight panelists. Rated as 1, not important, to 5, very important

Overall, panel members rated the sensitivity of a biopsy technique as the most important facilitator for the implementation of a biopsy technique. Furthermore, training of the radiologists is important, as experience in the use of biopsy techniques was rated as the second most important facilitator. For stereotactic and MRI-guided biopsies, the learning curves are quite short according to the panel, but for US-guided procedures, it is essential to obtain sufficient experience.

Furthermore, costs are an important barrier to the use of more expensive biopsy techniques, particularly VAB, and must be balanced against the likelihood of a better diagnosis.

Major complications requiring intervention are regarded as important, but as with any biopsy technique, are rare and, in general, not a reason to abstain from the use of a specific biopsy technique. Minor complications are important to consider for specific patient groups, such as adolescents, pregnant or lactating women. Although it is not considered an absolute contraindication, the use of VAB in lactating women is discouraged as, in rare cases, this may result in the formation of a fistula. The use of VAB, EVAB and VAE is technically more challenging when the lesion is situated close, less than 6 mm, to the dermis, pectoral muscle or nipple. In lesions close to the skin or nipple, anesthetic fluid or saline can be used to create sufficient space to avoid skin laceration.

Cosmetics, patient comfort, procedure time and patient factors were regarded as less important to consider. It was felt that obtaining a final diagnosis is usually more important and is not commonly influenced by these facilitators and barriers.

## Discussion

In this manuscript, an international expert statement is provided for the biopsy of breast lesions. While previous studies have focused on sensitivities and specificities of biopsy techniques a clear recommendation when to use what approach has been absent. Using a RAND/UCLA approach with interactive discussion on discrepantly scored items resulted in a general expert opinion of the optimal biopsy method for most lesions under most image-guiding modalities.

The panel agreed that ultrasound is, in principle, the guiding method of choice, due to procedural ease, flexibility of the approach, patient comfort, and real-time feedback. Other methods (e.g., DBT or MRI) are appropriate when the lesion is not visible under ultrasound. The lack of real-time feedback with other imaging methods, and the often subtle or diffuse nature of lesions without a clear ultrasound correlate led to the general statement that biopsy under imaging techniques other than ultrasound should be performed using VAB in all cases. Consequently, no further stratification on lesion types was attempted for guidance methods other than US. While data on biopsy accuracy with different methods is limited for other imaging techniques, such as CEM or dedicated breast CT, a few recent studies on CEM-guided biopsy provide valuable initial clinical insights. These studies reported early clinical experiences with CEM-guided biopsy in general using VAB, suggesting its potential utility [21–23]. However, further research is necessary to validate these results. It is acknowledged that not all hospitals will have the possibility to perform VAB under all imaging methods, and it is recognized that sometimes CNB under other modalities can give conclusive answers. However, when this is attempted, radiological-pathological correlation is essential, and in case of doubt, localization and surgical excisional biopsy should be performed.

Under ultrasound, various biopsy techniques are viable. Choice of method is, in essence, a balance between expected diagnostic accuracy, costs, and potential complications. Larger needles are, in general, more costly, with slightly more complications but higher diagnostic accuracy. Choosing a bigger needle is therefore appropriate in situations where the likelihood that a smaller needle might lead to failure of obtaining an accurate diagnosis is present. FNA is generally discouraged for breast interventional procedures, except for drainage of abscesses or symptomatic cysts. FNA requires extensive experience from both interventionalists and pathologists, and even then, the diagnostic accuracy is surpassed by histological biopsy techniques. CNB is the most important biopsy method under ultrasound due to the fact that most breast lesions appear as a mass that can be accurately targeted using real-time imaging. It should be noted that the 5 mm cut-off is somewhat arbitrary and should be regarded as an aid, rather than a hard threshold.

While, according to the panel, many specific ultrasound visible lesions can better be biopsied using VAB under ultrasound guidance than CNB, it should be realized that these lesions are less prevalent compared to masses. Still, the use of VAB under the guidance of ultrasound should be in the armamentarium of every breast interventionalist.

The panel felt the need to introduce a new term for vacuum-assisted biopsy taking multiple samples to obtain a reliable diagnosis, extended vacuum-assisted biopsy or EVAB. While in current literature these procedures are often grouped under VAE, this does not seem appropriate. The major difference between EVAB and VAE is that with EVAB, the intended goal remains to obtain an accurate diagnosis. Complete lesion removal is not the objective. As such, EVAB is a minimally invasive replacement of surgical excisional biopsy, aiming to exclude the presence of malignancy [24]. VAE, on the other hand, is reserved for situations where the actual aim is to fully remove a lesion. EVAB is usually performed after an initial smaller biopsy with inconclusive results and is particularly important in radiological-pathological discordance and the assessment of lesions of uncertain malignant potential. The panel majority preferred EVAB over surgical excision in all epithelial atypia. Surgical excision is a second-line option only in ADH, and should otherwise be reserved for phyllodes tumors and malignant lesions. The statements developed by a selected group of experts in this study, although focusing on biopsy method selection rather than diagnosis of specific lesions, are largely in line with existing international guidelines, including those published by the European Society of Breast Cancer Specialists (EUSOMA) [7] and the Swiss Society of Senology (SSS) [25], which provide specific recommendations for the management of high-risk lesions, also preferring minimal invasive strategies over surgical excision, albeit in the SSS recommendations the role of surgical excision biopsy is still larger. The panel felt that the diagnosis of DCIS in any biopsy is an indication for surgery, and additional biopsy is only indicated for staging if affecting treatment. However, it is acknowledged that several studies on watchful waiting in women with DCIS are ongoing [26–28]. If this proves safe, EVAB may be a viable method to exclude the presence of unsuspected invasive disease. Likewise, EVAB might be a potential method to assess the presence of residual disease in some breast cancer patients treated with neoadjuvant systemic therapy and a radiological complete response, but this also requires completion and analysis of ongoing studies [29–31].

VAE is regarded as a minimally invasive replacement of a complete excision of a lesion. This is mainly reserved for proven benign lesions up to 3 centimeters in size, albeit it is sometimes also possible for larger lesions, depending on the lesion position and the breast size, as well as the ease of the procedure, both for the practitioner and the patient. Recent data show that for small benign phyllodes tumors, VAE may also be a viable alternative to surgical excision. It should be noted that ultrasound-guided VAB of small lesions will often result in an (unintended) VAE. When histological evaluation does subsequently show a malignant breast lesion, the performed procedure does not replace a surgical excision, which should still be performed. Adequate marking of the biopsy site is therefore essential as the lesion might not be traceable after the procedure. There are several ongoing trials currently evaluating whether VAE might be feasible for small breast cancers, but results need to be awaited [32].

There are some limitations to this statement. The formulated advice is the result of discussion and voting within a small group of experts. Since numerical evidence on the best way to biopsy for every lesion is lacking, statements were formed according to classic evidence-based medicine, taking both the best available evidence and expert opinions and experiences into account. Future studies may increase the availability of numerical data and may potentially alter these statements slightly. While panel participants were recruited from across the world, not all continents, let alone countries, have been covered. Participants were asked to think from a global perspective and also bring forward the voices of their regional peers, but still, regional variability is possible, and local deviations from this general statement are likely to be present. This is partly due to variations in logistics and reimbursement policies across the world, and partly due to differences in the training and experience of practitioners. Local adaptation based on regional circumstances and resources may therefore be necessary. To ensure that the statements are directly applicable to clinical practice, participants were deliberately selected to include those professionals who are responsible for making biopsy decisions in routine care. In most regions worldwide, this responsibility predominantly lies with radiologists. Furthermore, while a literature review was performed to inform the discussions, this review was non-systematic, focusing on the most relevant and recent studies. It was not intended to be exhaustive or fully comprehensive. As a result, the statements rely on a combination of this selected evidence and the extensive clinical expertise of the expert panel.

Nonetheless, when in doubt, the panel feels that following these statements will lead to state-of-the-art care for patients with breast lesions that require biopsy, wherever in the world.

In conclusion, in the absence of clear guidelines regarding when to use what needle and approach for diagnostic breast biopsy under image guidance, standard approaches were formulated by an international expert group in order to provide handholds for everyday practice and ensure accurate diagnosis of all breast lesions.

## Supplementary information

ELECTRONIC SUPPLEMENTARY MATERIAL

## Abbreviations

ADH: Atypical ductal hyperplasia
CEM: Contrast-enhanced mammography
CNB: Core-needle biopsy
DCIS: Ductal carcinoma in situ
EVAB: Extended vacuum-assisted biopsy
FNA: Fine-needle aspiration
VAB: Vacuum-assisted biopsy
VAE: Vacuum-assisted excision
