# Supplementary material for: Image-guided biopsy of breast lesions—when to use what biopsy technique
Source: Insights Imaging. 2025 Sep 25;16:208. doi: 10.1186/s13244-025-02084-5 (PMC12463797; doi:10.1186/s13244-025-02084-5)
Supplement: Supplementary file 1 — ELECTRONIC SUPPLEMENTARY MATERIAL [file 13244_2025_2084_MOESM1_ESM.pdf]

Image guided biopsy of breast lesions – when to use what biopsy technique.

ELECTRONIC SUPPLEMENTARY MATERIAL

Content	Page
Appendix 1. Supplementary Table 2. Summary of the different imaging modalities used by the different panel members	2
Appendix 2. Summary of the introduction of the literature	3
Appendix 3. Voting questions	4
References	5

Appendix 1.

Supplementary Table 1. Summary of the different imaging modalities used by the different panel members

Purpose	Mammography	Ultrasound	MRI	CEM	Other (CT, PETCT)
Screening	100% (8/8)	62·5% (5/8)	75% (6/8)		
Diagnosis	87·5% (7/8)	100% (8/8)	87·5% (7/8)*	62·5% (5/8)	
Staging	50% (4/8)	62·5% (5/8)	87·5% (7/8)	50% (4/8)	12·5% (1/8)

* 1/8 only for high-risk lesions.

Appendix 2. Summary of the introduction of the literature

Fine needle aspiration (FNA): [1-3]

- Convenient
- Cost-effective
- High possibility of an inadequate specimen (3·5-11%)
- High rate of false negative (FN) findings (3-24%)
- Inability to distinguish IDC and DCIS
- Mainly indicated for cyst aspiration and metastatic lymph nodes not amenable to CNB

Core needle biopsy (CNB): [4-7]

- Lower rates of inadequate specimens (2-4%) than FNA
- Low rates of complications (0-3%)
- Initial data: US-guided CNB averts surgical excision in 85% with benign results with a decrease in 56% in the cost of diagnosis
- Initial data: US resulted in a single surgical procedure in 84% of women (compared to 29% of women undergoing surgical biopsy)
- Standard of care for biopsy in sonographically visible lesions
- Histologic underestimation due to insufficient tissue sampling (29% of breast lesions are heterogeneous)

Vacuum-assisted biopsy (VAB): [8-15]

- Safe and effective, no epithelial displacement, FNR 0-9%
- One single entry to obtain the necessary samples
- Lower rates of underestimation than CNB, x10 tissue volume
- Standard of care in MR-guided biopsies and stereotactic/tomo-guided biopsies for microcalcifications
- Larger size of the VAB probe → larger amount of local anaesthetic around the lesion
- Safe and real time control of the tip
- Reliable sampling of small masses or in case of a mismatch after CNB
- Capability of performing percutaneous excision of small fibroadenomas and abscesses as well as papillary lesions
- Higher amount of complications, bulky devices

Fundamental principles of US-guided VAB: [16]

- When lesions are visible, US guidance is the fastest, cheapest and most convenient guiding method available.
- Most common needle sizes 11G, 10G, 9G, 8G and 7G
- Number of cores per lesion and equivalences in VAB sizes → [11,12,17]

Radiological-pathological concordance [18]

The assessment of a successful vacuum-assisted breast biopsy depends on a partly or complete removal of the target lesion on imaging and a conclusive pathologic–radiologic correlation (concordance of histologic findings with imaging findings).

VAB meta-analyses: [19,20]

With high sensitivity (98%) and specificity (nearly 100%), VAB may provide a promising alternative for open breast biopsy and permit improved treatment planning.

CNB vs VAB cost analyses: [21]

When viewed from a health-systems' standpoint, vacuum- assisted breast biopsy is more cost-effective than 14-gauge spring-loaded core-needle biopsy for the diagnosis of breast lesions under ultrasound guidance.
When the cost of the biopsy procedure and surgery to evaluate high-risk, discordant, or progressing lesions are considered, health care payers spend less per cancer diagnosis whenthe initial biopsy is performed with a vacuum-assisted device.

Appendix 3. Voting questions

Panelists were asked to answer the following voting questions:

- Rate (on a five-point scale) and rank appropriateness of each biopsy method (FNA, CNB, VAB, EVAB, VAE and surgical excision) for general lesions visible on the following image guided techniques: US, mammography/tomosynthesis, CEM and MRI.
- What is the most appropriate biopsy method for the following lesion characteristics visible on US?
    - Mass > 5 mm
    - Mass < 5 mm
    - Simple/complicated cyst
    - Complex cystic and solid lesion with small (< 5 mm) solid part(s)
    - Complex cystic and solid lesion with larger (> 5 mm) solid part(s)
    - Intraductal mass (< 5 mm)/filled duct
    - Architectural distortion
    - Calcifications
    - Lymph nodes
    - Mastitis (not responding to antibiotic treatment)
    - Abscess
- What is the most appropriate re-biopsy method for the following pathology proven lesions visible on US?
    - Benign lesion that patient wants to be removed (< 3 cm)
    - Benign lesion that patient wants to be removed (> 3 cm)
    - Radiology-pathology discordance on CNB
    - Radiology-pathology discordance on VAB
    - B3 lesion on CNB
    - B3 lesion on VAB
        - Lobular neoplasia
        - Flat epithelial atypia
        - ADH
        - Complex sclerosing lesion
        - Papillary lesion
        - Benign phyllodes tumor
        - Borderline phyllodes tumor
    - DCIS on CNB
    - DCIS on VAB
- Rate (on a five-point scale) the importance of the following facilitators and barriers:
    - Sensitivity of the biopsy technique
    - Minor complications (no subsequent treatment needed)
    - Major complications (require intervention)
    - Cosmetics
    - Patient comfort
    - Procedure time
    - Costs
    - Possibility to remove the entire lesion
    - Experience of the radiologist
    - Patient factors (e.g. anticoagulation)

References

1. Fornage BD, Faroux MJ, Simatos A. Breast masses: US-guided fine-needle aspiration biopsy. *Radiology* 1987; **162**(2): 409-14.
2. Fornage BD. Interventional Ultrasound of the Breast; 2020.
3. Parker SH, Jobe WE, Dennis MA, et al. US-guided automated large-core breast biopsy. *Radiology* 1993; **187**(2): 507-11.
4. Liberman L, Feng TL, Dershaw DD, Morris EA, Abramson AF. US-guided core breast biopsy: use and cost-effectiveness. *Radiology* 1998; **208**(3): 717-23.
5. Liberman L, LaTrenta LR, Dershaw DD, et al. Impact of core biopsy on the surgical management of impalpable breast cancer. *AJR Am J Roentgenol* 1997; **168**(2): 495-9.
6. Morris EA, Liberman L, Trevisan SG, Abramson AF, Dershaw DD. Histologic heterogeneity of masses at percutaneous breast biopsy. *Breast J* 2002; **8**(4): 187-91.
7. Joshi M, Duva-Frissora A, Padmanabhan R, et al. Atypical ductal hyperplasia in stereotactic breast biopsies: enhanced accuracy of diagnosis with the mammotome. *Breast J* 2001; **7**(4): 207-13.
8. Simon JR, Kalbhen CL, Cooper RA, Flisak ME. Accuracy and complication rates of US-guided vacuum-assisted core breast biopsy: initial results. *Radiology* 2000; **215**(3): 694-7.
9. Youk JH, Kim EK, Kim MJ, Oh KK. Sonographically guided 14-gauge core needle biopsy of breast masses: a review of 2,420 cases with long-term follow-up. *AJR Am J Roentgenol* 2008; **190**(1): 202-7.
10. Schrading S, Strobel K, Keulers A, Dirrichs T, Kuhl CK. Safety and Efficacy of Magnetic Resonance-Guided Vacuum-Assisted Large-Volume Breast Biopsy (MR-Guided VALB). *Invest Radiol* 2017; **52**(3): 186-93.
11. Heywang-Kobrunner SH, Sinnatamby R, Lebeau A, et al. Interdisciplinary consensus on the uses and technique of MR-guided vacuum-assisted breast biopsy (VAB): results of a European consensus meeting. *Eur J Radiol* 2009; **72**(2): 289-94.
12. Heywang-Kobrunner SH, Schreer I, Decker T, Bocker W. Interdisciplinary consensus on the use and technique of vacuum-assisted stereotactic breast biopsy. *Eur J Radiol* 2003; **47**(3): 232-6.
13. Sperber F, Blank A, Metser U, Flusser G, Klausner JM, Lev-Chelouche D. Diagnosis and treatment of breast fibroadenomas by ultrasound-guided vacuum-assisted biopsy. *Arch Surg* 2003; **138**(7): 796-800.
14. Yom CK, Moon BI, Choe KJ, Choi HY, Park YL. Long-term results after excision of breast mass using a vacuum-assisted biopsy device. *ANZ J Surg* 2009; **79**(11): 794-8.
15. Carder PJ, Khan T, Burrows P, Sharma N. Large volume "mammotome" biopsy may reduce the need for diagnostic surgery in papillary lesions of the breast. *J Clin Pathol* 2008; **61**(8): 928-33.
16. Preibsch H, Baur A, Wietek BM, et al. Vacuum-assisted breast biopsy with 7-gauge, 8-gauge, 9-gauge, 10-gauge, and 11-gauge needles: how many specimens are necessary? *Acta Radiol* 2015; **56**(9): 1078-84.
17. Hahn M, Krainick-Strobel U, Toellner T, et al. Interdisciplinary consensus recommendations for the use of vacuum-assisted breast biopsy under sonographic guidance: first update 2012. *Ultraschall Med* 2012; **33**(4): 366-71.
18. Ho CP, Gillis JE, Atkins KA, Harvey JA, Nicholson BT. Interactive case review of radiologic and pathologic findings from breast biopsy: are they concordant? How do I manage the results? *Radiographics* 2013; **33**(4): E149-52.
19. Yu YH, Liang C, Yuan XZ. Diagnostic value of vacuum-assisted breast biopsy for]breast carcinoma: a meta-analysis and systematic review. *Breast Cancer Res Treat* 2010; **120**(2): 469-79.
20. Lu W, Tu L, Xie D, et al. A systematic review and meta-analysis: value of ultrasound-guided vacuum-assisted biopsy in the diagnosis and treatment of breast lesions. *Gland Surg* 2021; **10**(10): 3020-9.
21. Grady I, Vasquez T, Tawfik S, Grady S. Ultrasound-Guided Core-Needle Versus Vacuum-Assisted Breast Biopsy: A Cost Analysis Based on the American Society of Breast Surgeons' Mastery of Breast Surgery Registry. *Ann Surg Oncol* 2017; **24**(3): 676-82.
